# Water Vapor Sorption and Diffusivity in Bio-Based Poly(ethylene vanillate)—PEV

**DOI:** 10.3390/polym13040524

**Published:** 2021-02-10

**Authors:** Greta Giacobazzi, Claudio Gioia, Micaela Vannini, Paola Marchese, Valérie Guillard, Hélène Angellier-Coussy, Annamaria Celli

**Affiliations:** 1 Department of Civil, Chemical, Environmental, and Materials Engineering, Alma Mater Studiorum/University of Bologna, Via Terracini 28, 40131 Bologna, Italy; greta.giacobazzi2@unibo.it (G.G.); claudio.gioia2@unibo.it (C.G.); micaela.vannini@unibo.it (M.V.); paola.marchese@unibo.it (P.M.); 2 Agropolymers Engineering & Emerging Technology, University of Montpellier—INRAE-SupAgro, 2 Place Pierre Viala, Bât 31, 34060 Montpellier, France; helene.coussy@umontpellier.fr

**Keywords:** poly(ethylene vanillate), water sorption, water transport, diffusivity

## Abstract

The dynamic and equilibrium water vapor sorption properties of amorphous and highly crystalline poly(ethylene vanillate) (PEV) films were determined via gravimetric analysis, at 20 °C, over a wide range of relative humidity (0–95% RH). At low RH%, the dynamic of the sorption process obeys Fick’s law while at higher relative humidity it is characterized by a drift ascribable to non-Fickian relaxations. The non-Fickian relaxations, which are responsible for the incorporation of additional water, are correlated with the upturn of the sorption isotherms and simultaneously the hysteresis recorded between sorption and desorption cycles. The sorption isotherms of amorphous and highly crystalline PEV are arranged in the same concentration range of that of PET proving the similarity of the two polyesters. Water diffusion coefficients, whose determination from individual kinetic sorption/desorption curves required treatment with the Barens–Hopfenberg model, were demonstrated to be ≈10× higher for amorphous PEV compared to amorphous PET. Such a difference originates from the enhanced segmental flexibility of PEV chains.

## 1. Introduction

Lignin is the second most abundant polymer on earth (exceeded by cellulose) and the only renewable source of aromatics. Its availability at cheap prices, coupled with the need to replace petro-derived polymers, drives towards the development of renewable aromatic polymers [1]. Indeed, aromatic organic molecules, such as vanillic acid, ferulic acid, and sinapic acid, which could be obtained from a selective depolymerization of lignin, can be exploited in polymer science to produce new materials [2,3]. In such a framework, poly(ethylene vanillate) (PEV) is a new bio-based polyester developed from vanillic acid that is gaining attention because of its similarity to commercial poly(ethylene terephthalate) (PET). Both polyesters present a *para*-substituted aromatic ring and show similar thermal transitions. PEV is characterized by a glass transition temperature (*T_g_*) of 75 °C and a melting temperature (*T_m_*) of 264 °C [4] similarly to PET characterized by *T_g_* = 78 °C and *T_m_* = 252 °C [5]. Although a comprehensive study of this polymer in terms of mechanical performances is still missing, the aforementioned peculiarities make PEV a possible bio-derived substitute of PET as a packaging material.

Investigating the sorption and transport behavior of water vapor in polymeric materials is crucial for packaging applications, especially in food packaging. Indeed, water transfer through a packaging material may lead to detrimental effects on the food product, with loss of quality, accelerated degradation (through microbial development for instance) and reduction in food shelf life (e.g., remoistening of crispy products, dryness of soft or high a_w_ content foods, etc.). In addition, water sorption in the polymer matrix may deteriorate its mechanical and barrier properties (for instance O_2_ barrier properties) accentuating the degradation effect on food shelf life mentioned above.

In polymers, where the amorphous phase is in the glassy state at room temperature (*T_g_* > Troom temperature), water is known to plasticize the amorphous phase at high water vapor activity, resulting in a reduction in glass transition temperature [6] as well as the degradation of stiffness and strength [7]. At high temperature (near water boiling point) and high water vapor activity, water can hydrolyze polymers with COO, OH, NH groups, causing the rupture of the covalent bonds along the polymer backbone [7,8]. This leads to a reduction in molecular weight, which can be recorded as a glass transition temperature decrement. On the contrary, at low temperature (close to rt) even at high water vapor activity, such chemical degradation is improbable according to the literature and it is more likely that plasticization results from the rupture of the intermolecular weak hydrogen bonds, induced by swelling or water clustering [9].

Swelling is associated with the creation of strong water molecules–polymer interactions and a subsequent chain rearrangement, while water clustering results from the self-hydrogen bonding of water molecules when absorbed in a polymer, since water is a condensable penetrant. The swelling of the matrix and water clusters can, in turn, impact the diffusion of water vapor through the polymer and decrease or increase, respectively, their diffusion pathways. These effects are more pronounced in polyesters, since water shows great interaction with the ester groups [6].

The current study provides a detailed investigation of water vapor sorption kinetics in both amorphous and semicrystalline PEV at 20 °C using a gravimetric method.

PEV sorption data were compared to commercial amorphous PET data from the study of Dubelley et al. [10]. To understand the water sorption mechanism, the isotherms were modelled using a new dual mode sorption model, proposed by Feng [11]. This model, based on multilayer sorption theory and assuming the coexistence of dense polymer matrix and unrelaxed frozen microvoids, proves to successfully describe sorption in the investigated glassy polymers. To understand the water transport mechanism, the model proposed by Barens and Hopfeberg [12], which is a combination of Fickian diffusion and non-Fickian relaxations, was applied.

## 2. Materials and Methods

### 2.1. Materials

Poly(ethylene vanillate) (PEV) was synthetized in our laboratory, starting from methyl vanillate and ethylene carbonate trough an eco-friendly synthetic route developed by Gioia et al. [4,13].

Vanillic acid (VA) and ethylene carbonate (EC) with purities of 99% or more were purchased from Zentek, Milan, Italy. Potassium carbonate K_2_CO_3_ (99%) and antimony (V) oxide Sb_2_O_3_ (99%) were purchased from Sigma-Aldrich, Milan, Italy. Reagents were used as received without further purification during the synthesis. The synthetic procedure is reported in the Supplementary Materials.

The chemical structure of the synthetized polyester and its molecular weight were verified by ^1^H-NMR (Figure S2). The average molecular weight (*Mn*) was estimated at 11,000 g mol^−1^.

### 2.2. Films Preparation

The synthetized material was grinded using an Ika M20 Mill, Staufen, Germany. The resulting powder was dried at 60 °C for 24 h.

The semicrystalline film was obtained using a hydraulic thermo-press Carver, Wabash, Indiana, USA. Dried polymer powder was thermo-molded between two Teflon sheets at 275 °C for 2 min at 10 bar and 2 min at 100 bar. The film was quickly cooled to room temperature between the sheets under pressure by using running water.

The amorphous PEV film was obtained after quenching the molten sample in liquid nitrogen hindering crystallization.

The average thickness of the films was determined from 10 measurements randomly taken over the film surface using a precision thickness gauge, Hanatek instruments, St Leonards on Sea, East Sussex, UK and results to be about 600 μm.

### 2.3. Differential Scanning Calorimetry (DSC) Analysis

Differential scanning calorimetry (DSC) was used to study the thermal behavior and the degree of crystallinity of PEV films. Differential scanning calorimetry measurements were performed using a Q200 modulated DSC, equipped with a refrigerated cooling system TA Instruments, New Castle, DE, USA. For each experiment, the sample (around 5 mg) was placed in a hermetic aluminum pan and the measurement was performed under nitrogen atmosphere. Resulting thermograms display the variation of heat flow per grams of sample (W g^−1^) as function of temperature (°C).

The sample was first heated at 10 °C min^−1^ from 20 to 285 °C to study the degree of crystallinity of the film. Then it was cooled at 10 °C min^−1^ until −40 °C and finally heated at the same rate to 285 °C in order to study its thermal behavior.

### 2.4. Wide Angle X-ray Scattering (WAXS) Analysis

Wide angle X-ray scattering (WAXS) experiments were performed using an in-house setup of the Laboratoire Charles Coulomb, University of Montpellier, Montpellier, France. A high brightness low power X-ray tube coupled with an aspheric multilayer optic (GeniX3D from Xenocs) was employed. It delivers an ultralow divergent beam (0.5 mrad, λ = 0.15418 nm). Scatterless slits were used to give a clean 0.6 mm beam diameter with a flux of 35 Mphotons s^−1^ at the sample. We worked in a transmission configuration and scattered intensity was measured by a 2D “Pilatus” 300 K pixel detector by Dectris (490 × 600 pixels) with pixel size of 172 × 172 µm^2^, at a distance of 0.2 m from the sample. All intensities were corrected by transmission and the empty cell contribution was subtracted.

### 2.5. Dynamic Vapor Sorption/Desorption (DVS)

Kinetic gravimetric sorption measurements were performed using a controlled atmosphere Dynamic Vapor Sorption system (model DVS-1), Surface Measurements System Ltd., London, UK at 20 °C. This system provides a humidified air flow by mixing separate wet and dry air flows and the resulting steam passes over a pan containing the sample, which is attached to a Cahn microbalance. In this way, the DVS apparatus allows recording sample mass evolution with time as a function of relative humidity (RH). Mass evolution was carried out on circular samples of about 0.5 cm^2^ and 15 mg, deposited directly on the pan. It was considered as a plane sheet with diameter to half thickness ratio above 10 that is enough to neglect boundary effect [14,15]. Therefore, diffusion occurs from the upper side to the lower side, orthogonal in the thickness of the material without side effect. Before performing sorption/desorption kinetics experiments, the sample was dried at 0% RH in DVS chamber at 20 °C for 24 h to establish the dry mass. The sample was then exposed at 20 °C to the following RH profile: from 0% to 95% in increments of 10% up to 90% and 5% for the last point and from 95% to 0% in the same increments. The RH step lasted until reaching mass equilibrium. The sorption/desorption cycle was replicated twice on each sample to provide reproducibility. Undoubtedly, the reported material performances are representative for a real film for packaging applications.

Water sorption/desorption isotherms were determined from the equilibrium moisture uptake at each RH step. The equilibrium moisture uptake *m_uptake_* was used to determine the equilibrium water concentration at standard temperature and pressure *C* (cm^3^ STP cm^−3^ poly) in the polymer as follows:

(1) $$C=\frac{m_{uptake}\;\times \;V_{m}}{M_{w}\times V_{poly}}$$

where *V_m_* is the molar volume of water vapor (22,055 cm^3^ mol^−1^ at 20 °C), *M_w_* is the water molecular weight (18 g mol^−1^) and *V_poly_* is the sample polymer volume (cm^3^).

### 2.6. Modeling of Water Vapor Sorption Isotherm

Several mathematical models are proposed to describe the sorption isotherm behavior and determine the sorption parameters. In glassy polymers, the conventional dual mode sorption model (CDMS), which is a combination of the Langmuir mode sorption and Henry’s law, is largely applied to describe sorption isotherms at low water vapor activity until the isotherm exhibits concavity to activity axis [16]. However, the CDMS cannot effectively describe sorption isotherms, presenting an upturn at high a_w_ and it is thus inconsistent with the aim of the present work. The Guggenheim–Anderson–de Boer (GAB) model fits extremely well sigmoidal isotherms but the assumption of this model that all the sorption sites are equivalent is inconsistent with glassy polymers which are considered to have two species of sorption sites as the CDMS assumes: (i) the dense polymer matrix, and (ii) the non-equilibrium unrelaxed microvoids frozen in the glassy state [17,18,19].

Based on a multilayer sorption theory, on which the GAB equation is based and assuming the coexistence of two different sorption sites, as the CDMS does, Feng [11] proposed a new dual mode sorption model for water vapor sorption in glassy polymers able to describe concave, convex and sigmoidal isotherms. According to Feng, the sorbate concentration in the polymer is given as:

(2) $$C=C_{p}\frac{k^{\prime }a}{1-k^{\prime }a}+C_{p}\frac{\left(A^{\prime }-1\right)k^{\prime }a}{1+\left(A^{\prime }-1\right)k^{\prime }a}$$

where *a* is the water vapor activity, *C_p_* is the weighted mean value of the sorption capacity of a polymer to water; *k*′ is the ratio of the partition function of water vapor molecules sorbed in the multilayer (water–polymer interaction) to that of molecules in bulk liquid (water–water interaction); *A*′ is the ratio of the partition function of the first water vapor molecule sorbed on a microvoid to that of molecules sorbed beyond the first molecule in the multilayer. According to the assumption of the model, the interaction of a microvoid and the water molecules sorbed beyond the first molecule are equal to those of water-polymer. Therefore, *A*′ is a measure of the interaction of the water vapor molecule and a microvoid (water–micovoid interaction).

### 2.7. Diffusion in a Homogeneous Plane Sheet of Material

Water vapor diffusivity values were estimated for each relative humidity step from gravimetric water vapor sorption kinetic measurements. Water vapor sorption data can be easily converted to a non-dimensional form via Equation (3), useful for extracting diffusivity values:

(3) $$\frac{m_{t}}{m_{\infty }}=\left(\frac{m_{uptake}\left(t\right)}{m_{final}-m_{initial}}\right)$$

where *m_t_* denotes the water uptake at time *t* and *m_∞_* denotes the water uptake after infinite time represented by equilibrium since *m_initial_* is the measured mass at the beginning of the sorption step, *m_uptake_*(*t*) is the mass at time *t* and *m_final_* is the measured mass at the end of the sorption step.

Considering an isotropic diffusion problem in a plan sheet of material of thickness *L* (m), insulated at its bottom, the diffusion problem reduces to a pure monodirectional diffusion, well defined by Equation (4) given by Crank [20]:

(4) $${\frac{m_{t}}{m_{\infty }}}_{│C}=1-\overset{\infty }{\underset{n=0}{\sum }}\frac{8}{{\left(2n+1\right)}^{2}\pi^{2}}exp\left(-\frac{D\;{\left(2n+1\right)}^{2}\pi^{2}t}{L^{2}}\right)$$

where *D* (m^2^ s^−1^) is the Fickian diffusion coefficient averaged over the concentration interval. For more details about underlying equations, readers may refer to the work of Thoury-Monbrun et al. [15].

*D* is identified from transient data of each RH steps: therefore, one *D* value is obtained for each RH investigated. *D* is considered as the average value for full range; thus, *D* values reported in the present study are plotted at the midpoint of the respective sorption intervals.

The Fick model represented in Equation (4), however, fails to represent water transport mechanism when non-Fickian relaxations occur in addition to Fick diffusion. Barens and Hopfenberg [12] thus proposed a model which states that both Fickian diffusion (*F*) and relaxation processes (*R*) exist independently and can be combined using linear superposition, shown in Equations (5) and (6).

(5) $$m_{t}=m_{t,F}+m_{t,R}$$

(6) $${\frac{m_{t}}{m_{\infty }}}_{│BH}=\left[\phi \left({\frac{m_{t}}{m_{\infty }}}_{│C}\right)+\;\left(1-\phi \right)\left(1-exp\left(-\frac{1}{\tau_{R}}\right)\right)\right]$$

In Equation (5), *m_t,F_* represents the mass uptake ascribable to the Fickian diffusion (*F*), *m_t,R_* represents the mass uptake referable to non-Fickian relaxations (*R*) and *m_t_* is the total mass uptake at time *t* as sum of both mechanisms. In Equation (6), *φ* is a weighting factor that ranges from 0 to 1 and specifies the relative *F* (*φ* = 1) and *R* (*φ* = 0) contributions; *τ_R_* (s) is the time constant for the non-Fickian relaxations.

In the following sections, the subscript *C* denotes the Crank model, while the subscript *BH* denotes the Barens–Hopfenberg model.

### 2.8. Identification Parameter Procedure

Parameters of the water sorption isotherm and water vapor diffusivity were identified from MATLAB^®^ software solving a nonlinear least-squared fitting problem by *lsquonlin* function (Levenberg–Marquardt procedure). The *lsquonlin* MATLABroutine allows minimizing the root mean square error between experimental data *data_exp_* and the simulated data *data_sim_*, as follows:

(7) $$RMSE=\sqrt{\frac{\sum_{i=1}^{N}{\left((data_{sim}\left(i\right)-\;data_{exp}\left(i\right)\right)}^{2}}{N}}$$

where *N* is the number of terms in the predicted or experimental vector (*data_sim_* or *data_exp_*). According to Equation (7), the unit of the RMSE is the same as that of *data_sim_* or *data_exp_*.

To identify the parameters of water sorption isotherm Equation (2) was fitted onto experimental sorption curves.

To identify water vapor diffusivity, Equation (6) was fitted onto experimental kinetic sorption data. The fitting of Equation (6) to *m_exp_*(*t*) provides the identification of *D*, *φ*, and *τ_R_* at the same time. Due to the presence of three unknown parameters in Equation (6), caution should be exercised regarding the selection of the initial values required by the nonlinear least-squared fitting problem. To avoid any problem of local minimum, a range of different initial values of physical significance for each parameter (one at a time) was explored. The optimum is the set of values that is obtained each time with different initial values. As a starting point, to *D* was assigned an initial value previously identified by the Crank model (1 × 10^−11^ m^2^ s^−1^), to *φ* was assigned the value of 0.5 which is the average between the extremis 0 and 1, to *τ_R_* an initial value corresponding to the one calculated for amorphous PET in the study of Burgess et al. [21] (1 × 10^4^ s).

## 3. Results and Discussion

### 3.1. Chemical Structure and Thermal Transitions

**Figure 1:**
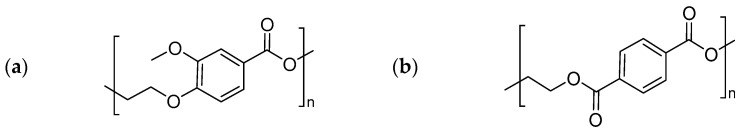
Chemical structures of (**a**) poly(ethylene vanillate) (PEV) and (**b**) poly(ethylene terephthalate) (PET).

In the current study, PEV was synthetized from methyl vanillate and ethylene carbonate through an eco-friendly synthetic route developed by Gioia et al. [4,13]. Its chemical structure (Figure 1a) is characterized by a *para*-substitute aromatic ring bonded to a carboxylic group from one side and to an ether group from the other. The benzoate structure also presents a methoxy group. The PEV chemical structure differs from that of PET (Figure 1b) in the substitution of a carboxyl group with an ether one and in the presence of an additional methoxy group.

**Figure 2:**
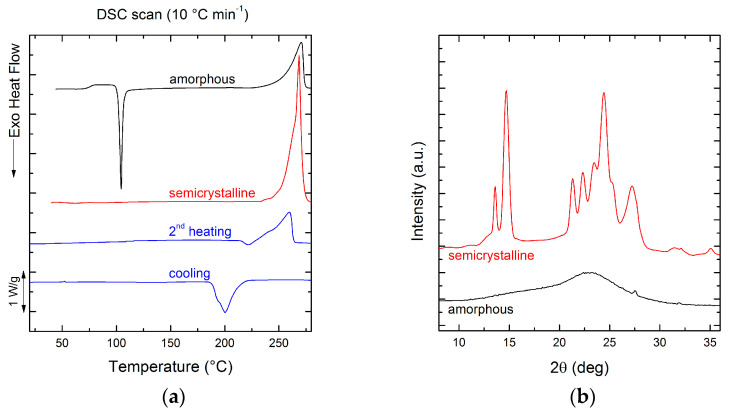
(**a**) Differential scanning calorimetry (DSC) first heating scans at 10 °C min^−1^ on amorphous and semicrystalline PEV films. DSC cooling and subsequent second heating scans at 10 °C min^−1^ on PEV after erasing the thermal history. (**b**) Wide angle X-ray scattering (WAXS) pattern on amorphous and semicrystalline PEV films.

As far as thermal behavior is concerned, PEV is characterized by a glass transition temperature (*T_g_*) of 75 °C and a melting temperature (*T_m_*) of 260 °C, as shown in Figure 2a and reported in Table 1. The thermal transition values are close to those of PET (*T_g_* = 78 °C and *T_m_* = 252 °C [5,22]), but the difference in the melting enthalpy (∆*H_m_*) between PEV (74 J g^−1^) and PET (45 J g^−1^) demonstrates the enhanced attitude of PEV to crystallize and to reach a state of high order. As stated by Gioia et al. [4] such behavior can be justified by considering a higher mobility of the constituent units of the PEV chain with respect to the terephthalic units of PET. Indeed, in terephthalic units, the sp^2^ hybridization of the carbon atoms of the two carboxylic groups induces coplanarity between carboxyl and phenyl groups, restricting the rotational angles of Cphenyl–COO to 0° and 180°. In PEV such rigidity is avoided because one carboxylic group is substituted by an ether unit, which can confer more flexibility to the chain and can impart a higher chain-folding capability, overcoming the hinderance of the lateral methoxy group.

### 3.2. Film Crystallinity

The structural state of amorphous and semicrystalline PEV films was determined by Differential Scanning Calorimetry (DSC) and Wide Angle X-ray Scattering (WAXS) analyses.

The first DSC heating scan of the quenched PEV film, obtained by melt-quenching in liquid nitrogen, is reported in Figure 2a. It is notable that the enthalpy of cold crystallization at 104 °C is quite identical to the enthalpy of melting at 271 °C, indicating that the film is in a completely amorphous state (∆*H_cc_* ≈ ∆*H_m_* = 76 J g^−1^). A second film of PEV was not quenched after compression-molding and the first DSC heating scan is reported in Figure 2a. The sample presents a single melting peak at 269 °C with a ∆*Hm* = 108.3 J g^−1^. By DSC analysis, the degree of crystallinity of the film was calculated as follows:

(8) $$X_{c}\;\left(\%\right)=\frac{\Delta H_{m}}{\Delta H_{m}^{{}^{\circ}}}\times 100$$

where ∆*H_m_* is the experimental melting enthalpy measured during the first heating ramp (J g^−1^) and $\Delta H_{m}^{{}^{\circ}}$ the theoretical melting enthalpy of a corresponding 100% crystalline sample. A value of $\Delta H_{m}^{{}^{\circ}}$ = 166 J g^−1^ was used according to the study of Zamboulis et al. [23] Therefore, from DSC analysis, the crystallinity turned out to be 65%, confirming the ability of PEV to reach a high degree of order. The crystallinity of the film was also calculated by WAXS analysis, whose spectrum is reported in Figure 2b. The degree of crystallinity was determined according to the formula [24]:

(9) $$X_{c}\;\left(\%\right)=\frac{I_{c}}{I_{c}+I_{a}}\times 100$$

where *I_c_* and *I_a_* are the intensities of X-rays scattered from the crystalline and the amorphous phases, respectively. The intensity of X-rays scattered from the crystalline regions of a specimen is proportional to the area under the sharp peaks of the spectrum, while the intensity of radiation scattered from the amorphous regions is proportional to the background area, which underlines the crystalline peaks. The intensity of radiation scattered from the entire sample (*I_c_* + *I_a_*) is proportional to the total area under the curve [25]. According to WAXS analysis, the degree of crystallinity is 60%. The value is quite close to that calculated by DSC analysis.

### 3.3. Sorption Measurements

**Figure 3:**
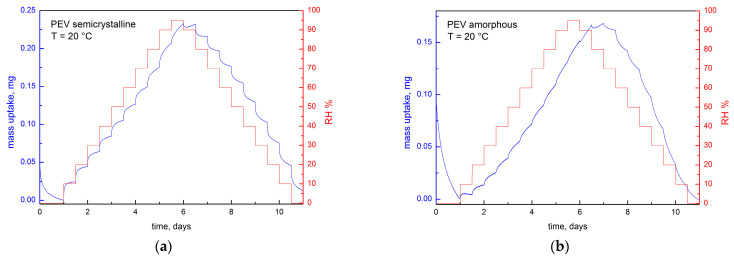
Example of gravimetric water vapor sorption/desorption kinetic measurements on (**a**) semicrystalline and (**b**) amorphous PEV (all measurements carried out at 20 °C).

The gravimetric water vapor sorption kinetic measurements on amorphous and semicrystalline PEV films were performed using a Dynamic Vapor Sorption (DVS) system at 20 °C over a wide range of relative humidity: from 0% to 95% in increments of 10% up to 90% and 5% for the last point, then followed by desorption measurements from 95% to 0% in same increments. Each RH step lasted 12 h. Examples of raw kinetic measurements are provided in Figure 3 for semicrystalline and amorphous PEV.

**Figure 4:**
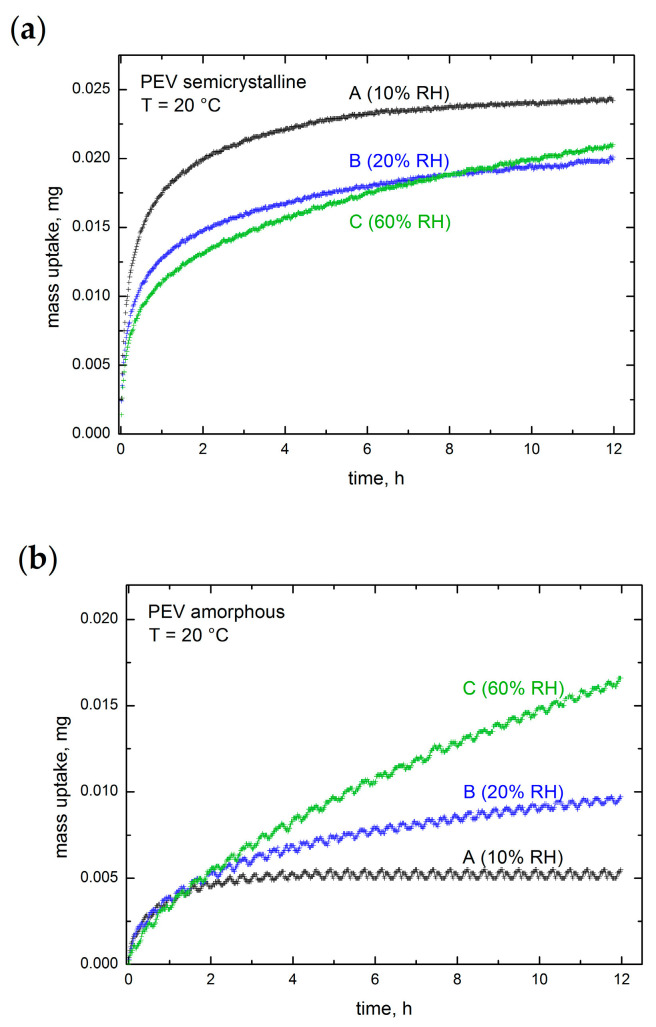

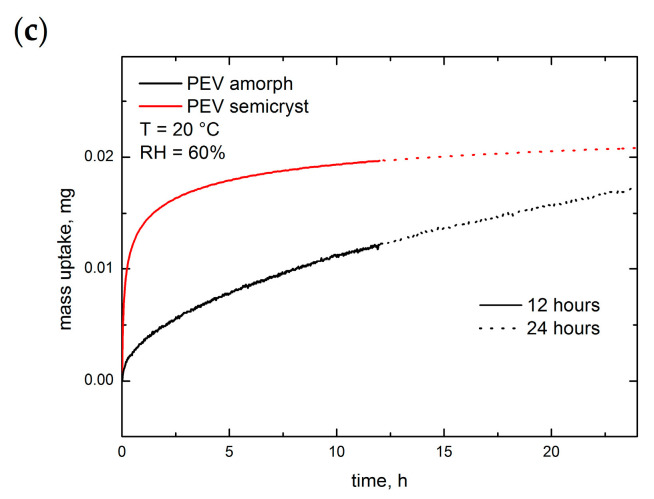
(**a**) Water vapor sorption kinetic of amorphous and (**b**) semicrystalline PEV at 20 °C and different relative humidity (10%, 20%, 60%). The time step is maintained constant at 12 h. (**c**) Water vapor sorption kinetic of amorphous and semicrystalline PEV at 20 °C and RH 60% for increasing the time step: 12 and 24 h.

Figure 4a,b show an excerpt of sorption kinetic at 10%, 20% and 60% RH for semicrystalline and amorphous PEV, respectively. At RH 10% and 20% (curves A and B in Figure 4a,b), the mass reaches the equilibrium after 12 h in both samples. This behavior relates to the random motion of vapor molecules without interaction with the polymer matrix: the water diffusion through the polymer is faster than water–polymer interaction and relaxation of the matrix, as it occurs when Fickian diffusion is predominating. On the contrary, at RH 60%, the mass equilibrium is not reached even after 12 h of exposition, as shown by curve C in both figures.

In order to investigate time-dependence at high water vapor activity a_w_, an extra experiment was conducted at constant temperature and RH (60%) by varying the time step. Both semicrystalline and amorphous PEV films were exposed at 20 °C to the following RH profile: from 50% to 60% RH for 12 h and 24 h time duration (Figure 4c). As shown, all the curves exhibit a mass drift, and the mass equilibrium is not reached even after 24 h of exposition at RH = 60%. This behavior has been largely described for glassy polymers in contact with condensed vapor and it was assigned to long-term non-Fickian relaxations of the polymer matrix causing an extra water vapor uptake without ever reaching mass equilibrium. For example, it is reported that poly(ethylene terephthalate) shows this drift of sorption curve at high water vapor activity (above a_w_ = 0.6), even at low temperature [10,21]. Several authors associated these relaxations in glassy polymers to morphological changes, such as plasticization [26,27] and free volume increase [21].

Since, at low temperature, the hydrolytic degradation of the covalent bonds is improbable, the authors associated free volume increase and plasticization to the swelling of the polymer matrix or water clustering.

### 3.4. Sorption Isotherms

Water vapor sorption isotherms were determined from the water vapor pseudo-equilibrium content at each RH% step. At each sorption measurement conducted by varying the RH, steady state must be reached for a correct interpretation of the sorption isotherm. Nevertheless, as discussed in the previous paragraph, mass equilibrium could not be achieved at high relative humidity because of the presence of non-Fickian relaxations. For semicrystalline PEV, relaxation is dominant from RH = 60%, while for amorphous PEV, sorption measurements have time-dependence from RH = 50%. As in the previous work of Barens et al. [12], we considered that the determination of the true equilibrium is impracticable, even increasing the step time duration and that the differences between the pseudo-equilibrium and the true equilibrium were minor due to the “extra” non-Fickian relaxations; therefore, all sorption measurements were stopped after 12 h of exposition.

**Figure 5:**
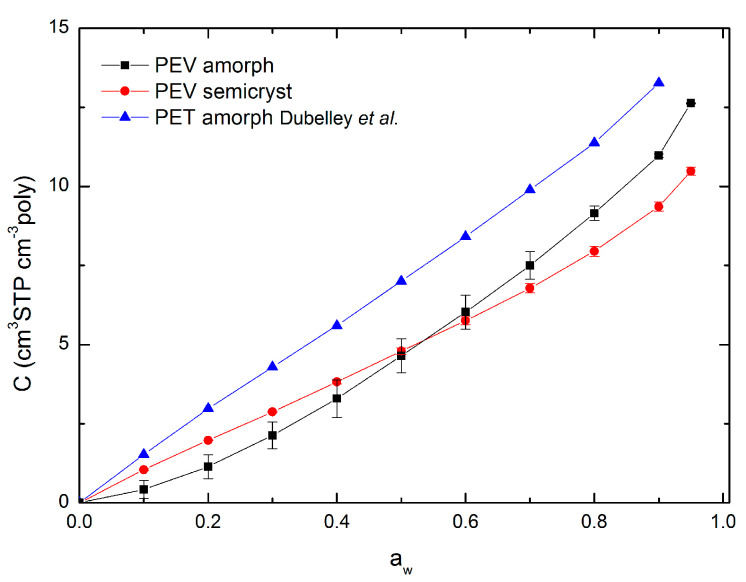
Water vapor sorption isotherms of amorphous and semicrystalline poly(ethylene vanillate) (PEV) at 20 °C (two replicates for each point). Water vapor sorption isotherm of amorphous PET at 23 °C from the study of Dubelley et al. [10] Lines are drawn to aid the eye and do not represents model fits.

Water vapor sorption isotherms of amorphous and semicrystalline PEV films in the water activity range between 0 and 0.95 at 20 °C are shown in Figure 5 (black and red full symbols, respectively). In the same Figure, the sorption isotherm of commercial amorphous PET (Rexor) from the study of Dubelley et al. [10] is reported in the water activity range between 0 and 0.90 at 23 °C (blue full symbols). As shown, the sorption isotherms of PEV and PET are arranged in the same concentration range, proving the similarity of the two polyesters.

The isotherm curves of semicrystalline PEV and amorphous PET are rather linear on the a_w_ range between 0 and 0.6 with a slight upturn for a_w_ > 0.6.

At low activity (a_w_ < 0.2) a concavity in the curve ascribable to the Langmuir mode sorption is generally observed: water molecules occupy specific sites (frozen microvoids) in the polymer and water–polymer interactions would predominate [28,29]. However, this concavity is difficult to observe in the case of semicrystalline PEV and amorphous PET. Between 0.2 and 0.6, a linear relation (*r*^2^ = 0.999 for semicrystalline PEV, *r*^2^ = 0.998 for amorphous PET) between the water concentration in the polymer and the water activity is noticed: the system water/polymer follows Henry’s law. In that later case, the penetrant is randomly dispersed in the matrix and polymer–polymer interactions are favored [30,31,32]. At high activity (a_w_ > 0.6) the curve registers an upturn: the water–polymer interactions become stronger, especially for semicrystalline PEV, for which this upturn is much more pronounced than for amorphous PET.

Contrary to semicrystalline PEV and amorphous PET, the isotherm curve of amorphous PEV is rather convex to the water activity axis on the entire a_w_ range indicating stronger water–polymer interactions. The upturn in the curve is also observed for a_w_ > 0.6.

The upward of the curves recorded at high a_w_ was already observed by some studies on PET and it has been related to swelling [21,33,34,35,36]. Therefore, it can be supposed that, for both amorphous and semicrystalline PEV, non-Fickian relaxations observed at high activity would result from swelling-induced plasticization of the polymer matrix. This should be confirmed by a deepened analysis of the diffusion phenomenon in the polymer.

**Figure 6:**
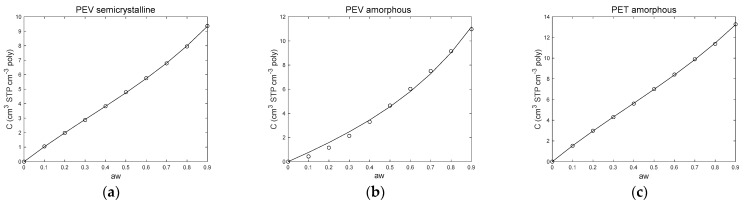
New dual mode sorption model fitting on (**a**) semicrystalline PEV water vapor sorption isotherm; (**b**) amorphous PEV water vapor sorption isotherm and (**c**) amorphous PET sorption data from the study of Dubelley et al. [10].

In the present work, the new dual mode sorption model (Equation (2)) is applied to amorphous and semicrystalline PEV and amorphous PET [10] sorption isotherms in the water activity range 0–0.90. Figure 6 shows the new dual mode sorption model fitting and Table 2 lists the three parameters *C_p_*, *k*′ and *A*′ of the model. A very good fitting of experimental sorption data was obtained for amorphous PET and semicrystalline PEV with very low root mean square error (RMSE) value (less than 0.05 cm^3^ STP cm^−3^ poly) while a less good, but still acceptable, fit was obtained for amorphous PEV (RMSE value of 0.28 cm^3^ STP cm^−3^ poly). Therefore, the sorption curves of semicrystalline PEV and amorphous PET are well fitted by the new dual mode sorption model confirming that, even if obviously almost linear, the curves rather display a sigmoidal pattern.

The parameter *C_p_* (cm^3^ STP cm^−3^ poly) follows the order amorphous PET > amorphous PEV > semicrystalline PEV, confirming that PET has higher sorption capacity than both amorphous and semicrystalline PEV samples. It is well known that water molecules interact with carboxylic groups on the polyester backbone via hydrogen bonding, and the lower sorption capacity of PEV can be related to the carboxylic groups’ concentration on the polymer backbone, which is lower for PEV than PET because of the substitution of one carboxylic group with an ether group from one side. The increased sorption capacity of amorphous PEV, compared to semicrystalline PEV, was expected since crystallites act as impermeable particles in the polymer matrix [37].

The parameter *k*′ respects the trend: amorphous PEV > semicrystalline PEV > amorphous PET. The mathematical meaning of *k*′ is to describe the departure from linearity of the isotherm curve. The greater the upturn at high water activity, the larger the value of *k*′, meaning stronger water–polymer interactions. As previously discussed, at high water vapor activity, the swelling-induced plasticization of the polymer matrix is hypothesized and, according to the new dual mode sorption model, the *k*′ value is an indication of the water molecule–polymer interactions, which lead to such a phenomenon. By comparing amorphous and semicrystalline PEV samples, the parameter *k*′ is higher for amorphous PEV, meaning that, in semicrystalline PEV, the water–polymer interactions responsible for swelling are less important than in amorphous PEV. Since swelling occurs in the amorphous phase, this result was expected. Moreover, PEV is demonstrated to have a higher *k*′ value than PET, meaning that chain rearrangement is more pronounced in the new bio-based aromatic polyester.

Finally, the parameter *A*′ ≠ 1 for all the samples, confirming that the polyesters contain microvoids and, thus, are in their glassy state.

### 3.5. Hysteresis

Sorption hysteresis occurs when the penetrant sorption and the subsequent desorption cycles do not superimpose and can occur in a wide variety of penetrant–polymer combinations. Very often, hysteresis is linked to the swelling of the polymer matrix, where the chains irreversibly relax because of the rupture of intermolecular hydrogen bonds and incorporate extra penetrant at high concentration [38,39].

**Figure 7:**
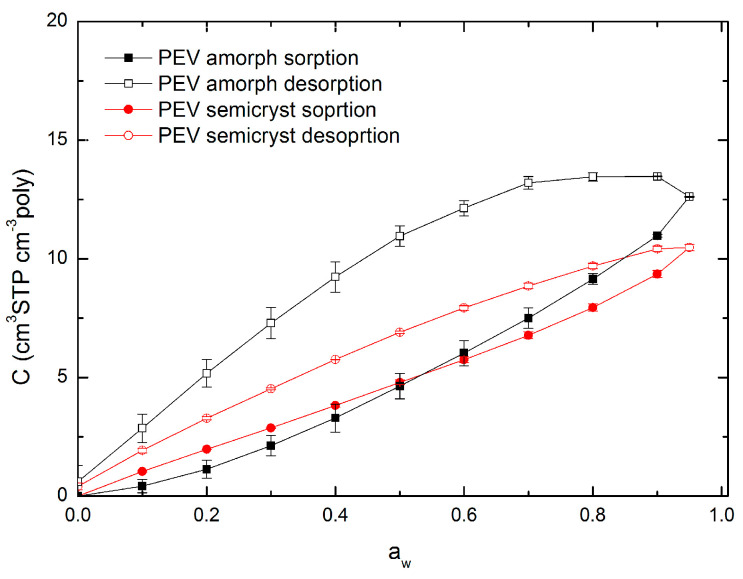
Sorption hysteresis at 20 °C for amorphous and semicrystalline PEV (two replicates for each point). Lines are drawn to aid the eye and do not represents model fits.

Figure 7 shows that water uptake in amorphous and semicrystalline PEV exhibits hysteresis between sorption and subsequent desorption cycles. This behavior correlates with the upturn in concentration vs. the activity of the sorption isotherm (Figure 5) and the presence of non-Fickian relaxations and demonstrates the occurrence of morphological changes in the polymer matrix because of the swelling-induced plasticization. Hysteresis is much more important for amorphous PEV than for semicrystalline PEV, confirming the higher swelling of the polymer matrix in such a sample film. The present observation will be deeply discussed in a diffusivity chapter (Section 3.6).

### 3.6. Diffusivity

Water vapor diffusivity values were estimated for each relative humidity step from individual gravimetric water vapor sorption kinetic measurements.

Individual kinetic sorption/desorption uptake curves obtained for one full cycle in DVS and modelled with the BH model are provided as examples of raw data in Figure S3A for amorphous PEV and in Figure S4A for semicrystalline PEV. The residuals between experimental sorption kinetic data *m_exp_* and the simulated ones *m_sim_* according to the BH model fit are also reported in Figures S3B and S4B for amorphous and semicrystalline PEV, respectively.

**Figure 8:**
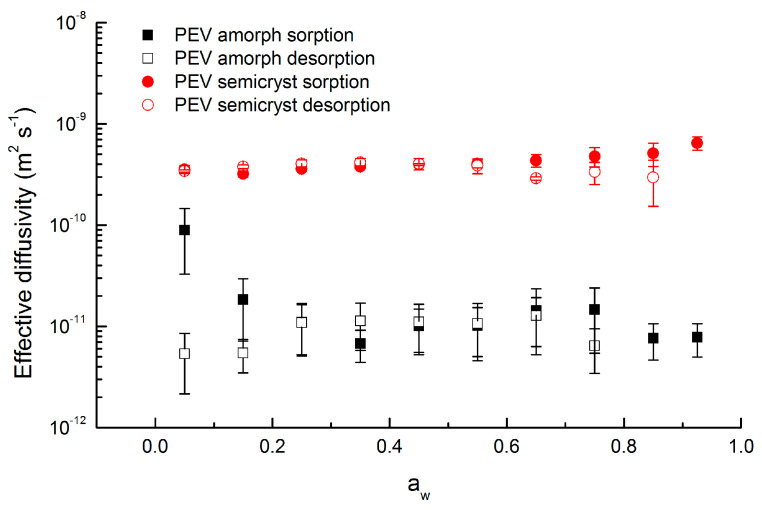
Effective water vapor diffusivity (*D_eff_*) at 20 °C in amorphous and semicrystalline PEV determined from BH model (two replicates for each point). Sorption and subsequent desorption values are plotted at the midpoint of the respective activity sorption interval.

**Figure 9:**
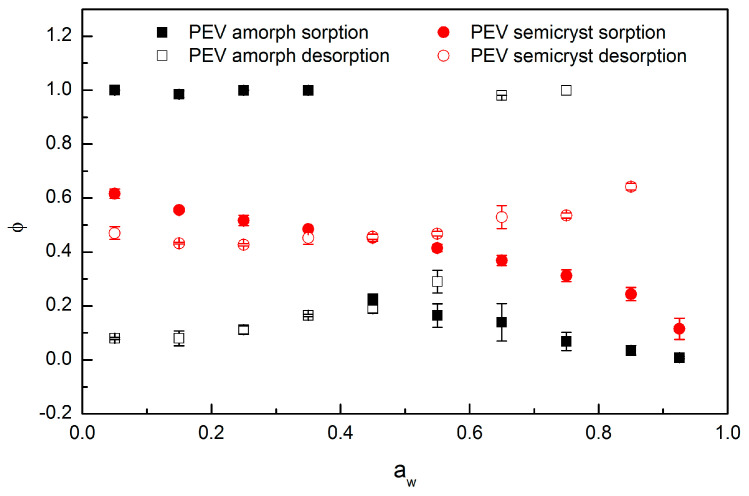
Plot of *φ* from the BH model for water vapor at 20 °C in amorphous and semicrystalline PEV for sorption and desorption (two replicates for each point).

**Figure 10:**
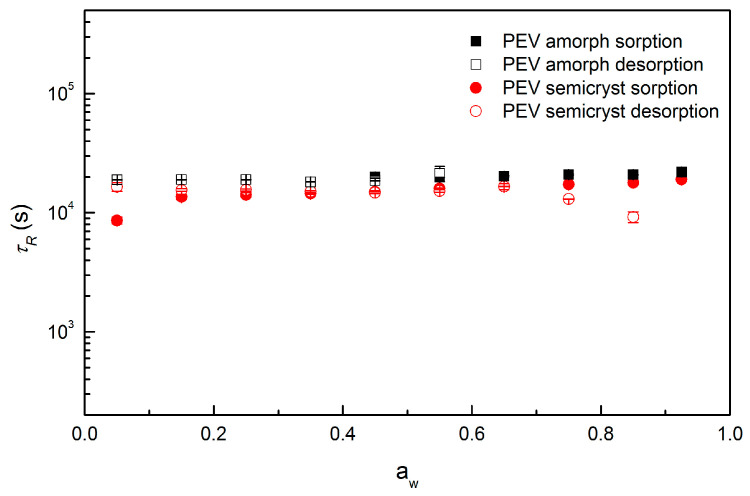
Plot of time of relaxation (*τ_R_*) from the BH model for water vapor at 20 °C in amorphous and semicrystalline PEV for sorption and desorption (two replicates for each point).

The parameters *D* (m^2^ s^−1^), *φ* and *τ_R_* (s) obtained from the BH model fit to the kinetic sorption/desorption curves of amorphous and semicrystalline PEV are plotted in Figure 8, Figure 9 and Figure 10, respectively.

Since *D* is an overall mass transport phenomenon that does not only represent Fick’s diffusion, but all the different water transport mechanisms occurring in the material, *D* is conceived as an apparent or effective diffusivity, noted as *D_eff_* in the following.

As shown in Figure 8, in both amorphous and semicrystalline samples, in spite of few variations, it may be considered that diffusivity remains almost constant over the entire water vapor activity range investigated. In addition, the values for sorption and desorption are similar in magnitude over the a_w_ range. Therefore, an average diffusivity value was calculated over the entire water vapor activity range investigated: *D_eff_* = 4.0 × 10^−10^ m^2^ s^−1^ for semicrystalline PEV and 1.0 × 10^−11^ m^2^ s^−1^ for amorphous PEV.

Semicrystalline PEV shows a *D_eff_* value significantly higher than amorphous PEV and the explication has to be found in the different degree of crystallinity. In semicrystalline polymers, the crystalline regions act as impermeable barriers analogous of filler particles. However, in highly crystalline materials the permeable amorphous phase is trapped in between a crystal and the near one and thus, the interface amorphous/crystalline constitutes a considerable fraction of the sample. Bastioli et al. [7] reported how water sorption in the amorphous phase of a semicrystalline polymer can produce stress at the amorphous/crystalline interface, followed by “microvoids” formation that might favor penetrant diffusion and this is the case of semicrystalline PEV (Xc = 60%).

As concerns amorphous PEV, it exhibits an average *D_eff_* value of 1.0 × 10^−11^ m^2^ s^−1^ across the entire concentration range. The *D_eff_* value of amorphous PEV is higher than the *D_eff_* value of commercial amorphous PET reported in literature. Dubelley et al. [10] determined a value of *D_eff_* equal to 6.1 × 10^−13^ m^2^ s^−1^ for water diffusion at 23 °C after a correction of swelling on amorphous PET (Rexor); Burgess et al. [21] observed an average *D_eff_* value of 1.5 × 10^−12^ m^2^ s^−1^ at 35 °C by using the BH model on amorphous PET supplied by Coca-Cola.

By comparing PEV and PET chemical structures, the PEV aromatic ring is less sterically hindered by the presence of an ether group from one side rather than two carboxylic groups, as for PET. As a consequence, the aromatic ring has a greater range of rotation, which leads to an enhanced segmental flexibility of the chain which can result in favored chain rearrangement when water molecules are absorbed. The greater diffusion coefficient for PEV, compared to PET, originates from the inherent differences in segmental mobility.

A plot of parameter *φ*, which is a weighting factor that specifies the relative Fickian (*φ* = 1) and relaxation (*φ* = 0) contributions vs. water activity is provided in Figure 9 for amorphous and semicrystalline PEV.

For semicrystalline PEV in sorption mode, the contribution ascribable to relaxation process is evident for the entire a_w_ range, even at low water vapor activity (*φ* always < 1) and the inversion from Fickian-dominated to relaxation-dominated process occurs at 0.55 of water activity. However, after 0.55 of water activity, the contribution of the relaxation-dominated process, even predominant, remains low (*φ* about 0.4/0.5 except for a_w_ > 0.85). The parameter *φ* of amorphous PEV levelled off at 1 in sorption mode until a_w_ = 0.35, then drops suddenly below 0.2 and stays low up to a_w_ = 0.925. At a_w_ < 0.35, the diffusion in amorphous PEV is clearly Fickian and quickly reversed to pure relaxation mechanism after 0.35. This trend is close to those of amorphous PET and PEF at 35 °C as observed by Burgess et al. [21]. However, for both PET and PEF, the inversion from Fickian to relaxation occurs at 0.6 instead of 0.35 water activity, meaning that PEV would start relaxing at lower a_w_. The rotation of the aromatic ring and the presence of the lateral methoxy group determine an enhanced segmental mobility of the PEV polymer backbone, which results in favored chain rearrangement when water molecules are absorbed.

In semicrystalline PEV, the relaxation phenomenon is evident even at low water vapor activity because the absorption of water occurs in the rigid amorphous phase, which is the amorphous region trapped in between a crystal and the near one [40]. As demonstrated by Lin et al. [41], this rigid amorphous fraction surrounding the crystallites is less dense than the core of the amorphous phase, resulting in enhanced sorption capability and the eased rupture of intermolecular hydrogen bonds. Nevertheless, above 0.55 activity, the *φ* value remains higher for semicrystalline than for amorphous PEV because the volume fraction occupied by crystallites remains unaffected during the entire sorption process and the relaxation is ascribed only to the amorphous phase. The lower the volume fraction of the amorphous phase, the lower the impact of plasticization over the sample.

As concerns desorption, for semicrystalline PEV, Fickian diffusion is dominant during the entire activity range, since *φ* ≥ 0.5, while for amorphous PEV, Fickian diffusion is dominant until 0.65 activity; afterwards, relaxation-dominated processis predominant. In desorption mode, the protracted approach to equilibrium, recorded as *φ* ≠ 1, can result from the collapse of extra free-volume introduced during sorption, as observed by Barens et al. [42] in the case of vinyl chloride sorption in poly(vinyl chloride).

The plot of *τ_R_* vs. activity is provided in Figure 10 for both amorphous and semicrystalline PEV. The parameter *τ_R_* is determined accurately for the kinetic uptake curves for whose *φ* is far from 1. Consequently, Figure 10 only reports τ_R_ values in conjunction with the respective *φ* parameters represented in Figure 9.

Both amorphous and semicrystalline PEV samples exhibit similar relaxation rates despite their different degree of crystallinity and the values are very close to those of PET and PEF at 35 °C as observed by Burgess et al. [21] in their study of water vapor kinetic sorption. Therefore, relaxation-dominated process in both amorphous and semicrystalline PEV is detected as *φ* ≠ 1 and times of relaxation of about 2 × 10^4^ s.

## 4. Conclusions

The water transport mechanism in amorphous and semicrystalline PEV demonstrates compliance to Fickian-type sorption at low water vapor activity. On the contrary, at high water vapor activity, the water vapor sorption curves exhibit a mass drift due to the presence of non-Fickian relaxations and mass equilibrium is not reached. The sorption isotherms were determined from pseudo-equilibrium mass content and modelled using a new dual mode sorption model. Amorphous PEV exhibits slightly higher water uptake compared to semicrystalline PEV, since crystallites act as impermeable filler particles in the polymer matrix. Compared to amorphous PET, amorphous PEV exhibits lower water uptake due to the lower concentration of carboxylic groups on the polyester backbone. The irreversible relaxation of PEV polymer chains results in the incorporation of additional water at high activity, which is recorded as the upturn of the sorption isotherm, and simultaneously in a hysteretic behavior during desorption. The relaxation of polymer chains can be related to swelling-induced plasticization. Because of the presence of non-Fickian relaxations, the determination of water diffusion coefficient from individual kinetic sorption/desorption uptake curves required treatment with the Barens–Hopfenberg model. Semicrystalline PEV exhibits a significantly higher water diffusion coefficient compared to the completely amorphous sample because the stress at the amorphous/crystalline interface produced by water absorption in the amorphous phase induces “microvoids” formation that favors water diffusion. As concerns amorphous PEV, it exhibits higher averaged water diffusion coefficient of ≈10× compared to amorphous PET. With respect to PET, the PEV aromatic ring is less sterically hindered by the presence of an ether group from one side rather than two carboxylic groups and thus the ring-flipping is not restricted to defined angles of rotation leading to an enhanced segmental flexibility of the chain. The greater diffusion coefficient for PEV compared to PET originates from the inherent differences in segmental mobility.

## Figures and Tables

**Table 1:** Calorimetric data obtained from Differential Scanning Calorimetry (DSC) scans at 10 °C min^−1^. The enthalpies have been normalized by the weight fraction of the samples.

	*T_c_* (°C)	∆*H_c_* (J g^−1^)	*T*_cc_ (°C)	∆*H*_cc_ (J g^−1^)	*T_m_* (°C)	∆*H_m_* (J g^−1^)	*T_g_* (°C)
1st heating amorph ^1^	-	-	104	75.8	271	77.3	75
1st heating semicryst ^2^	-	-	-	-	269	108.3	-
2nd heating ^3^	-	-	-	-	260	74.2	-
Cooling ^3^	200	72.8	-	-	-	-	-

^1^ First heating scan on melt-quenched film; ^2^ first heating scan on compression-molded film; ^3^ cooling and second heating scans after erasing the thermal history.

**Table 2:** Sorption parameters *Cp*, *k*′, *A*′ of amorphous and semicrystalline PEV and amorphous PET according to the new dual mode sorption model.

Sample	a_w_ Range	Cp (cm^3^ STP cm^−3^ poly)	*k*′	*A*′	RMSE (cm^3^ STP cm^−3^ poly)
PEV amorph	0–0.9	7.757	0.605	1.502	0.282
PEV semicryst	0–0.9	6.661	0.528	3.087	0.029
PET amorph Dubelley	0–0.9	10.161	0.496	3.217	0.049

## Data Availability

The data presented in this study are available on request from the corresponding author.

## Supplementary Materials

The following are available online at https://www.mdpi.com/2073-4360/13/4/524/s1, Figure S1: ^1^H-NMR spectrum of methyl vanillate. Figure S2: ^1^H-NMR spectrum of poly(ethylene vanillate) (PEV). Figure S3: (A) Kinetic sorption/desorption data for water vapor on amorphous PEV at 20 °C measured by DVS. Red lines represent model fits from the BH model while experimental data are represented in blue. (B) Root mean square errors between experimental sorption kinetic data *m_exp_* and the simulated ones *m_sim_* according to BH model fit. Figure S4: (A) Kinetic sorption/desorption data for water vapor on semicrystalline PEV at 20 °C measured by DVS. Red lines represent model fits from the BH model while experimental data are represented in blue. (B) Root mean square errors between experimental sorption kinetic data *m_exp_* and the simulated ones *m_sim_*, according to BH model fit.
