# Supplementary material for: Water Vapor Sorption and Diffusivity in Bio-Based Poly(ethylene vanillate)—PEV
Source: Polymers (Basel). 2021 Feb 10;13(4):524. doi: 10.3390/polym13040524 (PMC7916482; doi:10.3390/polym13040524)
Supplement: Supplementary file 1 [file polymers-13-00524-s001.pdf]

Water vapor sorption and diffusivity in bio-based poly(ethylene vanillate) – PEV

Greta Giacobazzi, Claudio Gioia, Micaela Vannini, Paola Marchese, Valérie Guillard, Hélène Angellier-Coussy and Annamaria Celli

Supplementary materials

Synthesis of methyl vanillate monomer

Vanillic acid (33.6 g, 0.200 mol), methanol (300 ml) and sulfuric acid (2 ml) were introduced in a round-bottom flask equipped with a magnetic stirrer and a reflux column. The mixture was heated at 80 °C for 24 hours until a clear solution was obtained. Methanol was removed under reduced pressure; the product was solubilized in 75 ml of ethyl acetate and washed twice with deionized water (100 ml). The organic phase was collected, anhydrified and ethyl acetate was removed under reduced pressure to obtain a dense oil. Finally, the product was crystallized in water to obtain pure methyl vanillate (yield 98%).

*^{1}H-NMR (400 MHz, $CDCl_3$, δ): 7.63 (dd, J_1=1.95 J_2=8.2 Hz, 1H, C^cH), 7.55 (d, J=1.95 Hz, 1H, C^bH), 6.94 (d, J=8.2 Hz, 1H, C^dH), 3.94 (s, 3H, C^aH_3), 3.88 (s, 3H, C^eH_3) ppm (Figure S 1).

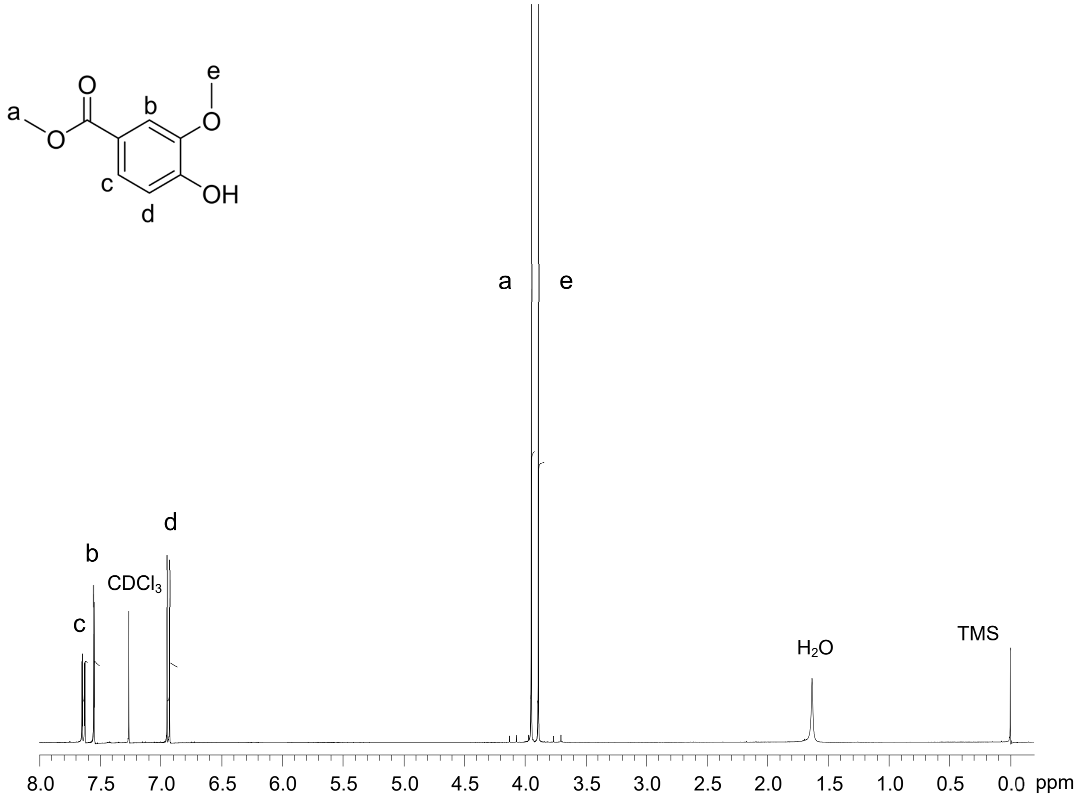

Figure S 1. ^{1}H-NMR spectrum of methyl vanillate.

Synthesis of poly(ethylene vanillate) (PEV)

Methyl vanillate (25.0 g, 0.137 mol), ethylene carbonate (13.3 g, 0.151 mol), K_2CO_3 (0.142 g, 0.00103 mol), Sb_2O_3 (1 wt% to the final product) were introduced into a 500ml glass reactor bottle equipped with a mechanic stirrer and connected to nitrogen flow. The polycondensation was performed in two stages. In the first stage, the mixture was heated under nitrogen atmosphere at 180 °C for 1 hour and then the temperature was gradually increased until 240 °C in the subsequent 2 hours. In the second stage, the pressure was slowly reduced from atmospheric to 0.3 mbar while the temperature was gradually increased up to 270 °C during 3 hours. The resulting material was collected from the reactor without purifications (yield 80%).

*^{1}H-NMR (400 MHz, $CDCl_3$/CF_3COOD, δ): 7.74 (dd, 1H, C^cH), 7.61 (d, 1H, C^bH), 6.99 (d, 1H, C^dH), 4.76 (t, 2H, C^gH_2), 4.48 (t, 2H, C^fH_2), 3.93 (s, 3H, C^eH_3) ppm (Figure S 2).

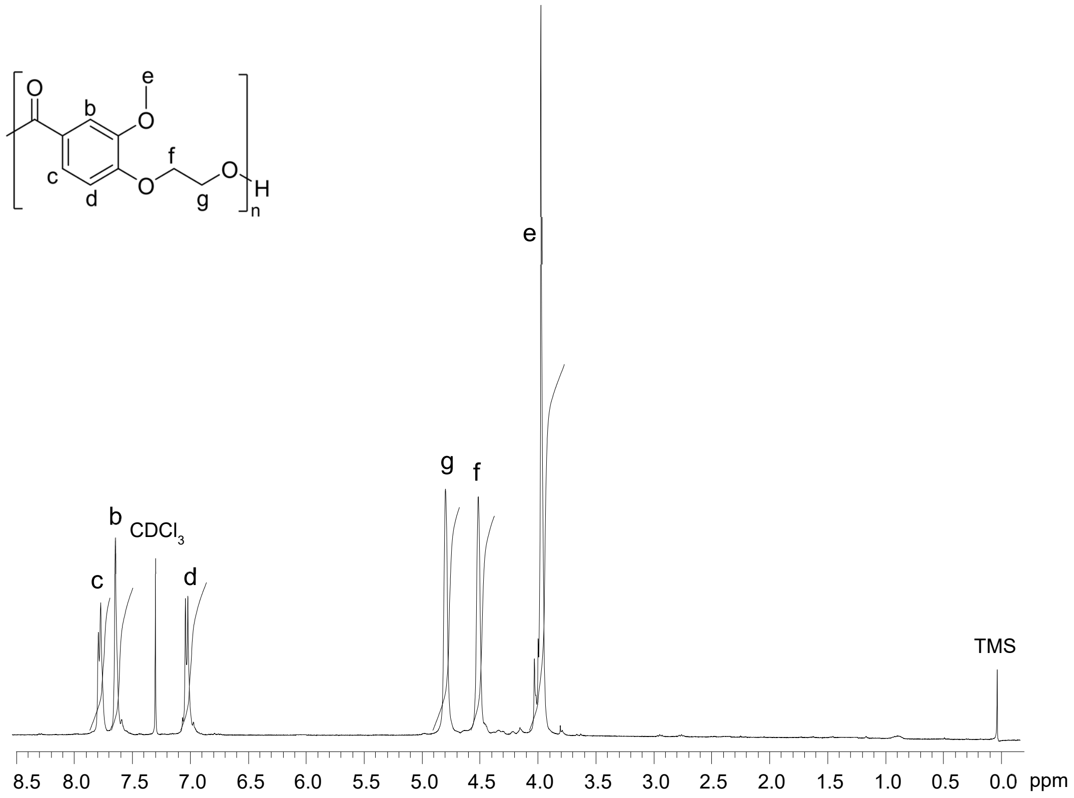

Figure S 2. ^{1}H-NMR spectrum of poly(ethylene vanillate) PEV.

* ^{1}H-NMR spectra were recorded at room temperature on samples dissolved in $CDCl_3$ or $CDCl_3/CF_3COOD$ (80/20 v/v) mixture using a Varian Mercury 400 spectrometer, the proton frequency being 400 MHz. The measurements were performed at 25 °C. Chemical shifts (δ) are reported in part per million with reference to chloroform solvent ($CDCl_3$).

Individual kinetic sorption/desorption uptake curves

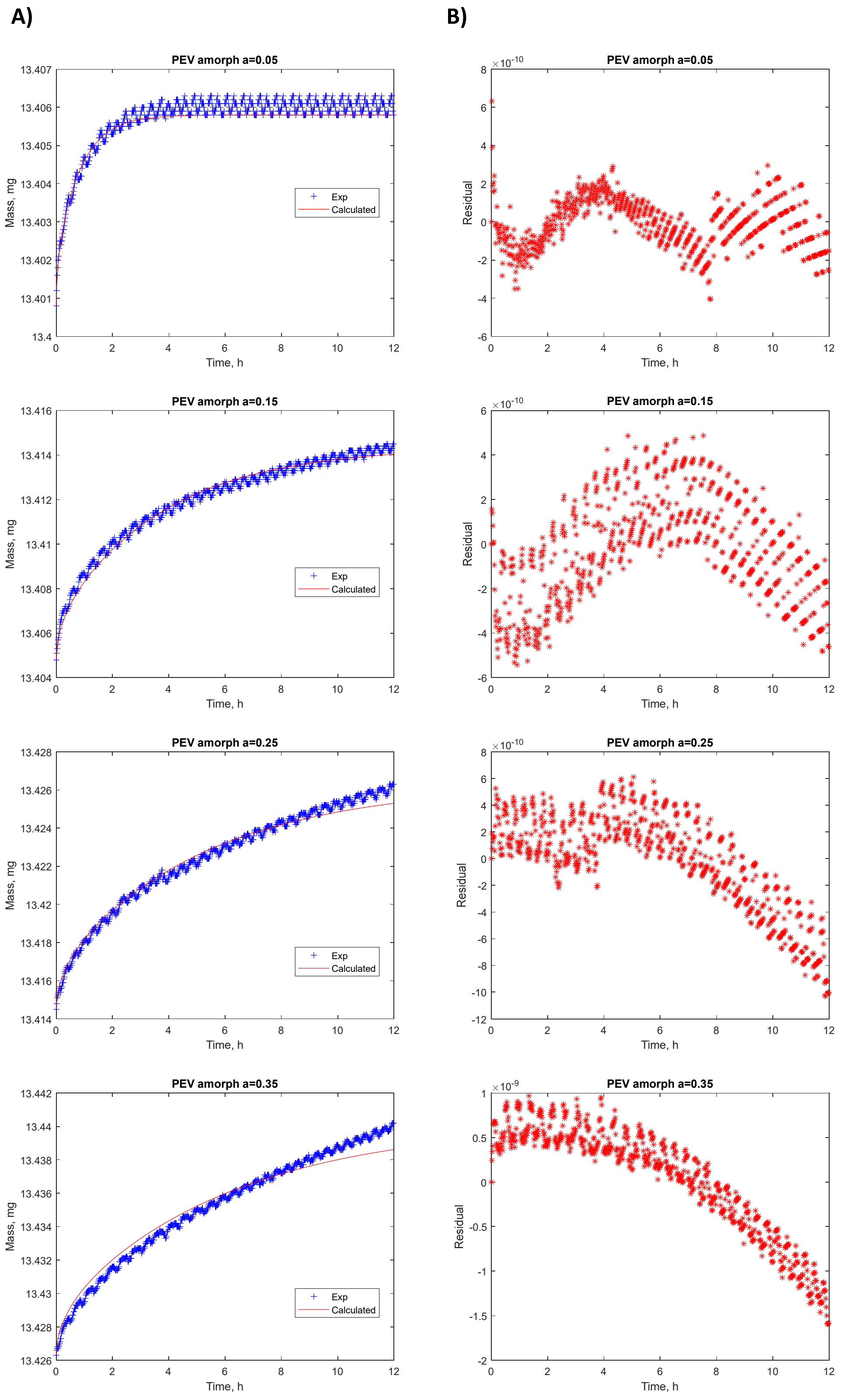

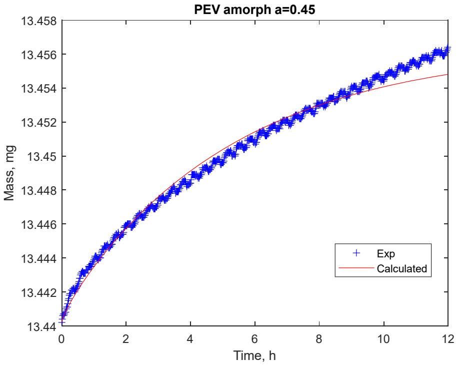
PEV amorph a=0.45
Mass, mg
Time, h
Exp
Calculated

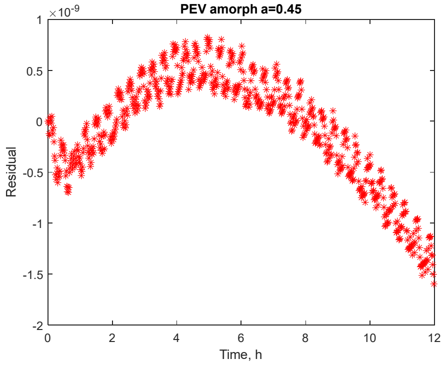
PEV amorph a=0.45
×10-9
Residual
Time, h

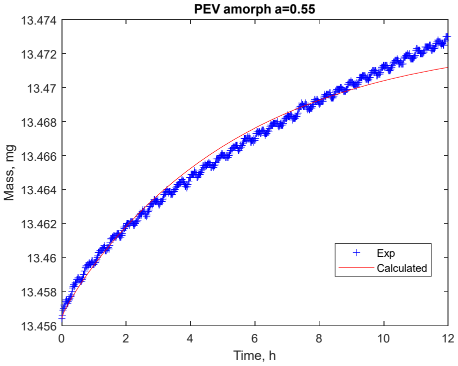
PEV amorph a=0.55
Mass, mg
Time, h
Exp
Calculated

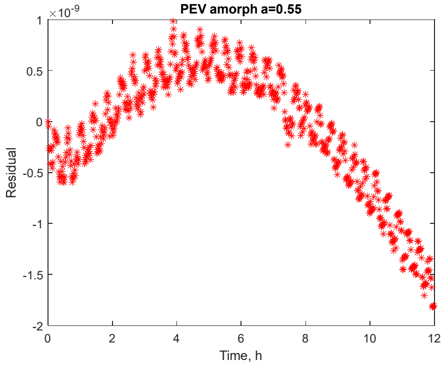
PEV amorph a=0.55
×10-9
Residual
Time, h

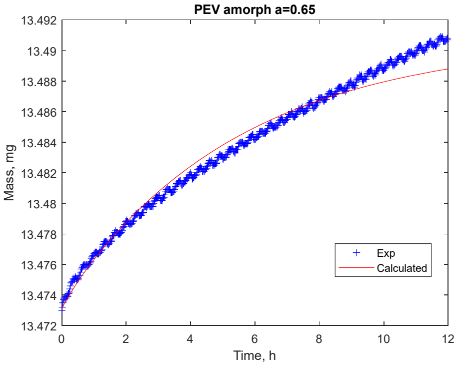
PEV amorph a=0.65
Mass, mg
Time, h
Exp
Calculated

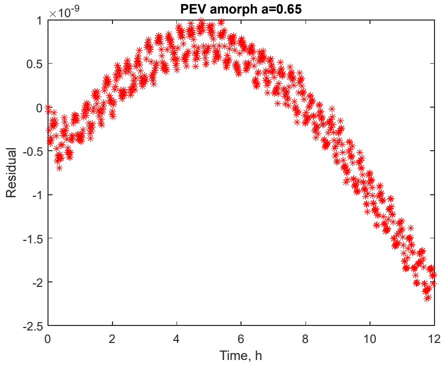
PEV amorph a=0.65
×10-9
Residual
Time, h

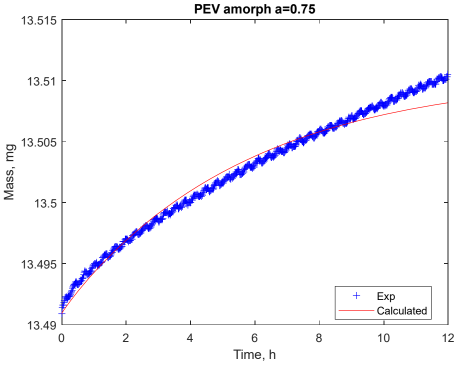
PEV amorph a=0.75
Mass, mg
Time, h
Exp
Calculated

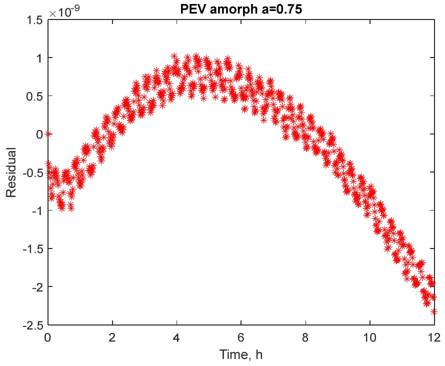
PEV amorph a=0.75
×10-9
Residual
Time, h

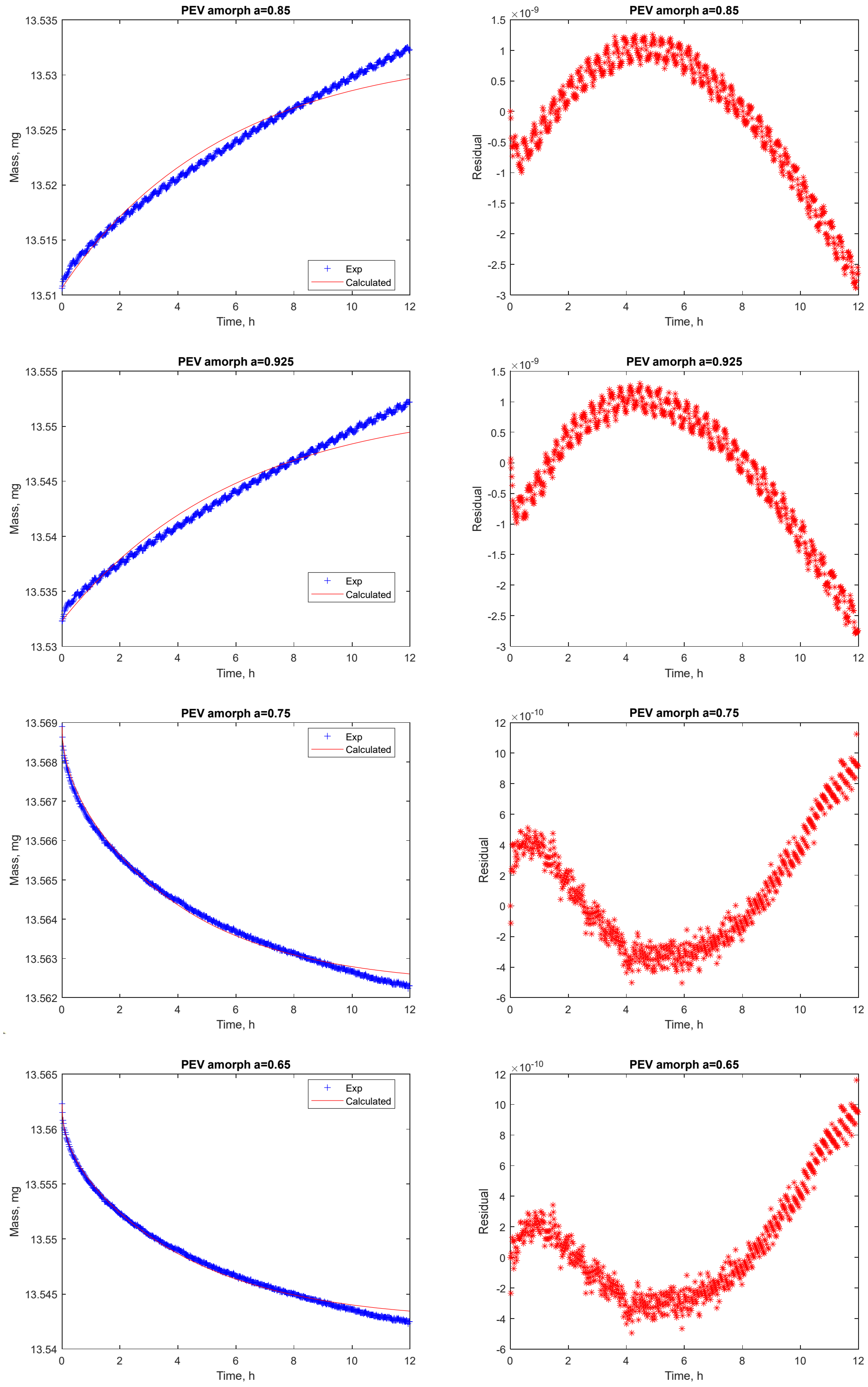
PEV amorph a=0.85
Mass, mg
Time, h
Exp
Calculated
PEV amorph a=0.85
Residual
Time, h
×10^-9
PEV amorph a=0.925
Mass, mg
Time, h
Exp
Calculated
PEV amorph a=0.925
Residual
Time, h
×10^-9
PEV amorph a=0.75
Mass, mg
Time, h
Exp
Calculated
PEV amorph a=0.75
Residual
Time, h
×10^-10
PEV amorph a=0.65
Mass, mg
Time, h
Exp
Calculated
PEV amorph a=0.65
Residual
Time, h
×10^-10

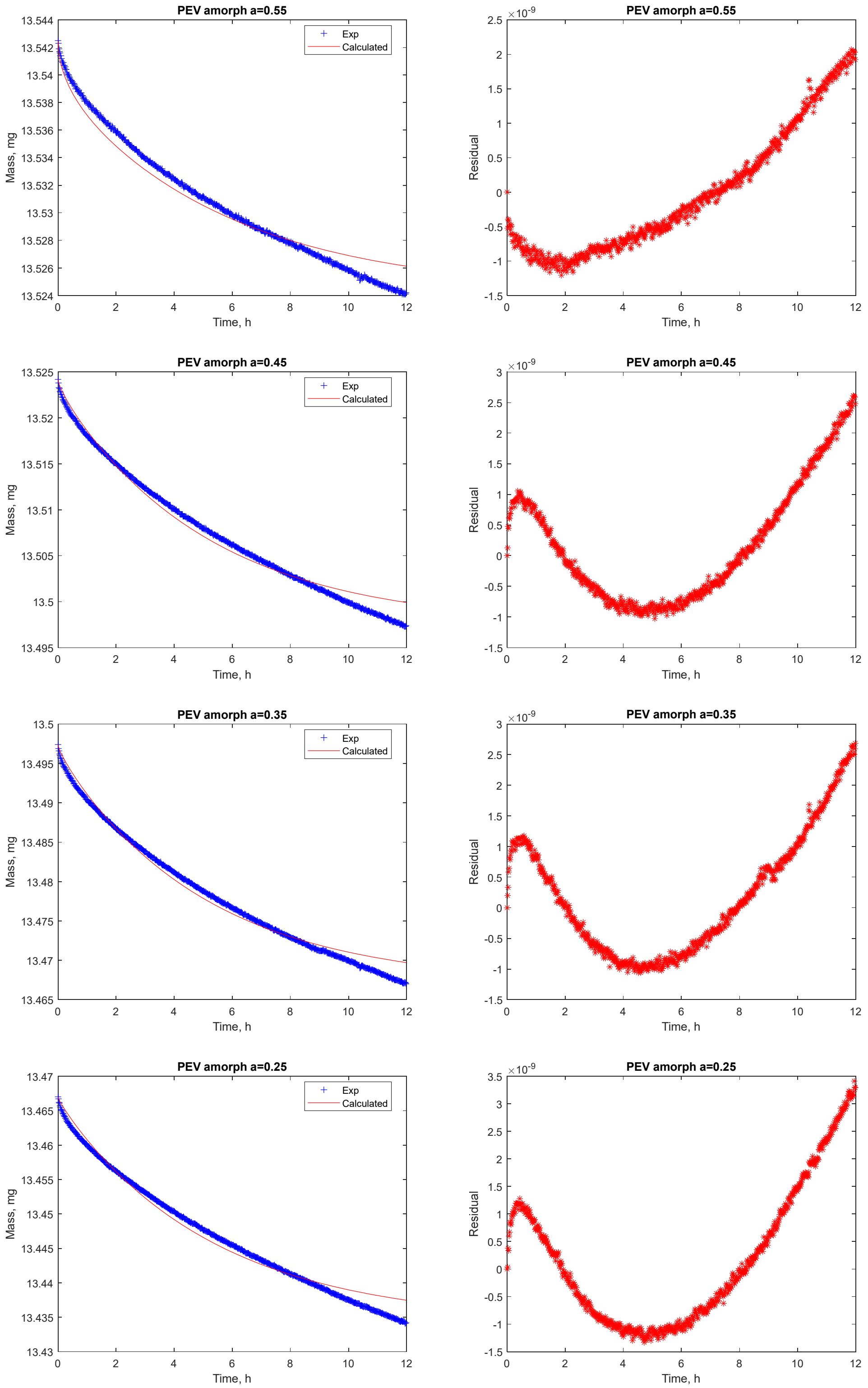
PEV amorph a=0.55
Mass, mg
Time, h
Exp
Calculated
Residual
PEV amorph a=0.45
PEV amorph a=0.35
PEV amorph a=0.25

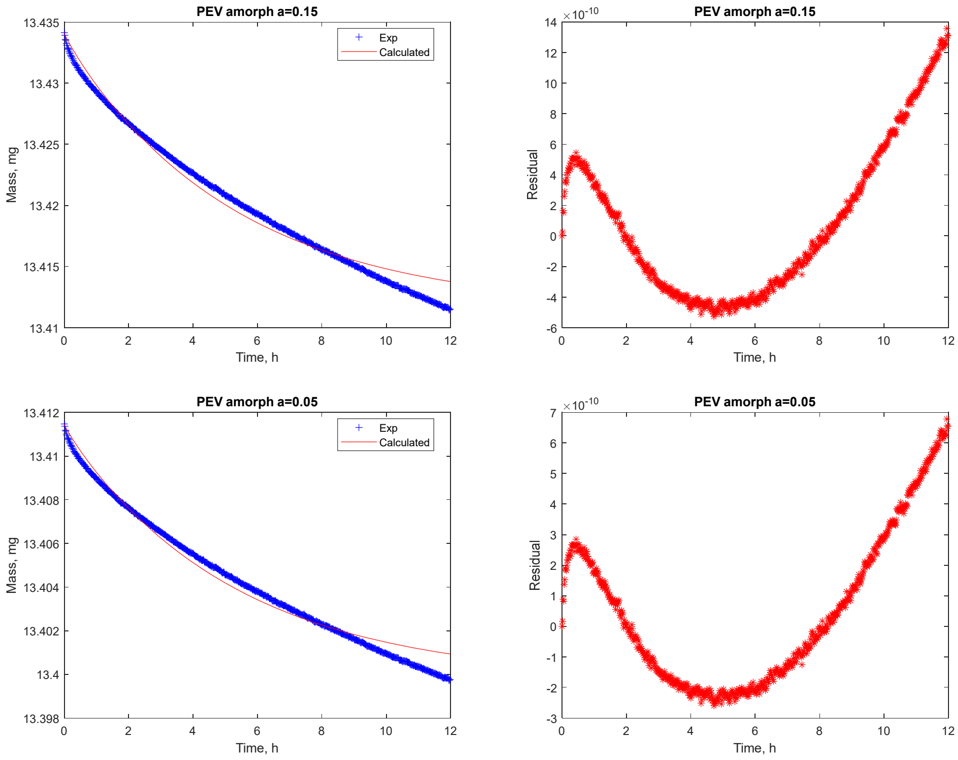

Figure S 3. A) Kinetic sorption/desorption data for water vapor on amorphous PEV at 20 °C measured by DVS. Red lines represent model fits from the BH model while experimental data are represented in blue. **B)** Root mean square errors between experimental sorption kinetic data m_{exp} and the simulated ones m_{sim} according to BH model fit.
The sorption intervals are labelled in each respective graph.

A) **B)**

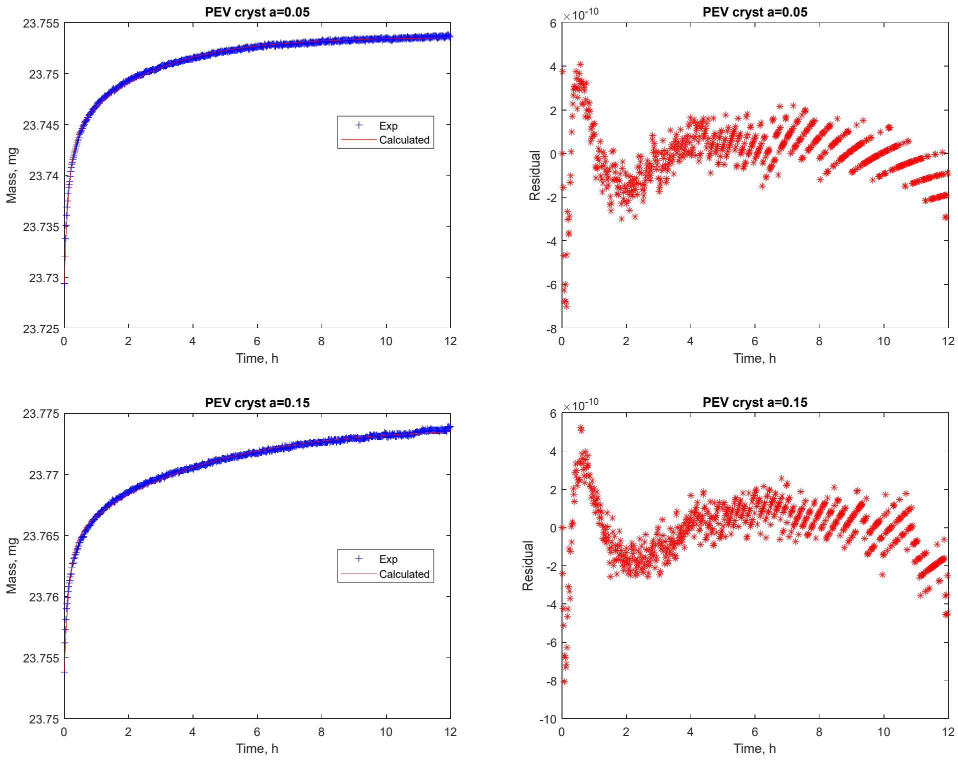

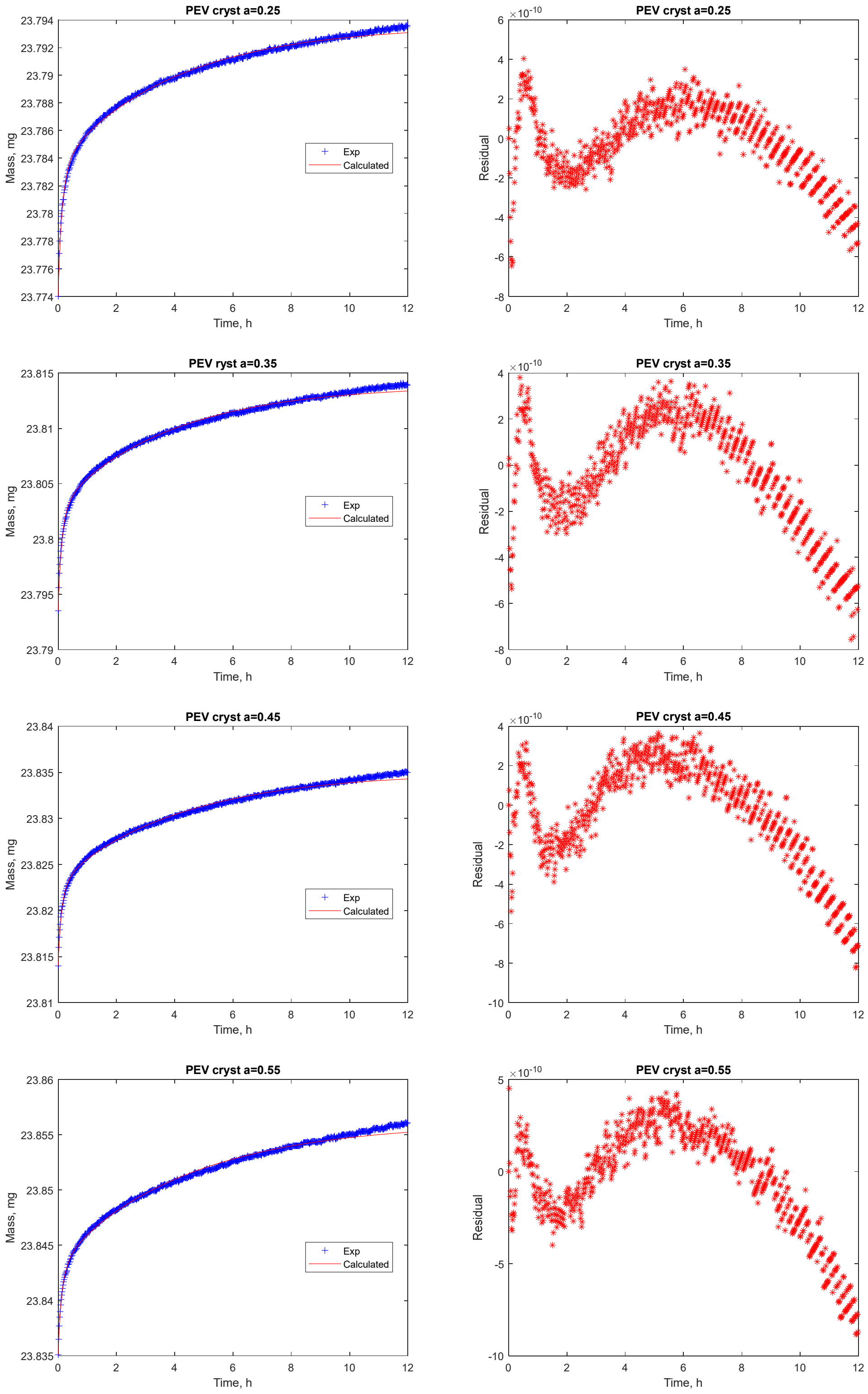
PEV cryst a=0.25
Mass, mg
Time, h
Exp
Calculated
PEV cryst a=0.25
Residual
×10^-10
Time, h
PEV ryst a=0.35
Mass, mg
Time, h
Exp
Calculated
PEV cryst a=0.35
Residual
×10^-10
Time, h
PEV cryst a=0.45
Mass, mg
Time, h
Exp
Calculated
PEV cryst a=0.45
Residual
×10^-10
Time, h
PEV cryst a=0.55
Mass, mg
Time, h
Exp
Calculated
PEV cryst a=0.55
Residual
×10^-10
Time, h

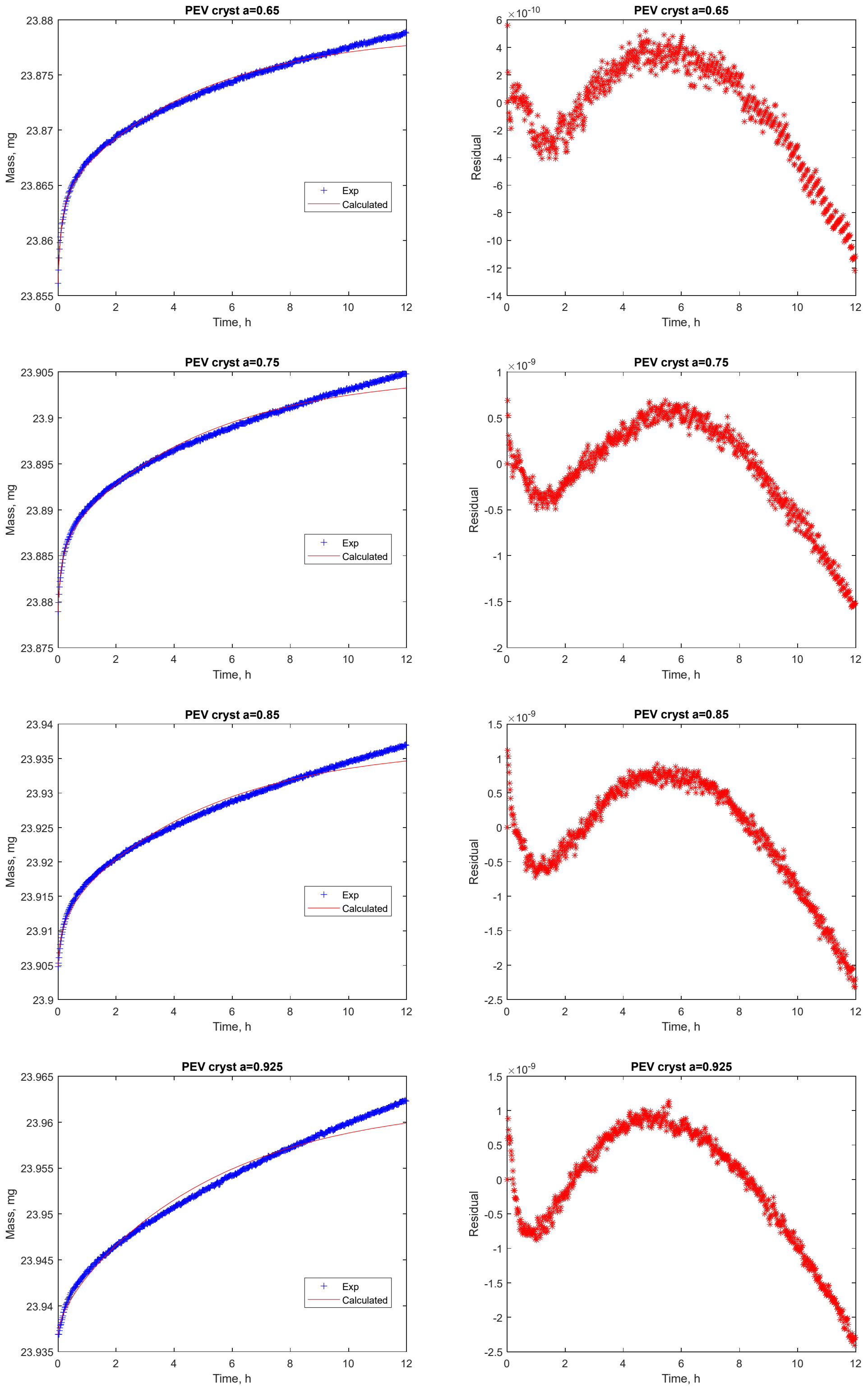
PEV cryst a=0.65
Mass, mg
Time, h
Exp
Calculated
PEV cryst a=0.65
Residual
Time, h
PEV cryst a=0.75
Mass, mg
Time, h
Exp
Calculated
PEV cryst a=0.75
Residual
Time, h
PEV cryst a=0.85
Mass, mg
Time, h
Exp
Calculated
PEV cryst a=0.85
Residual
Time, h
PEV cryst a=0.925
Mass, mg
Time, h
Exp
Calculated
PEV cryst a=0.925
Residual
Time, h

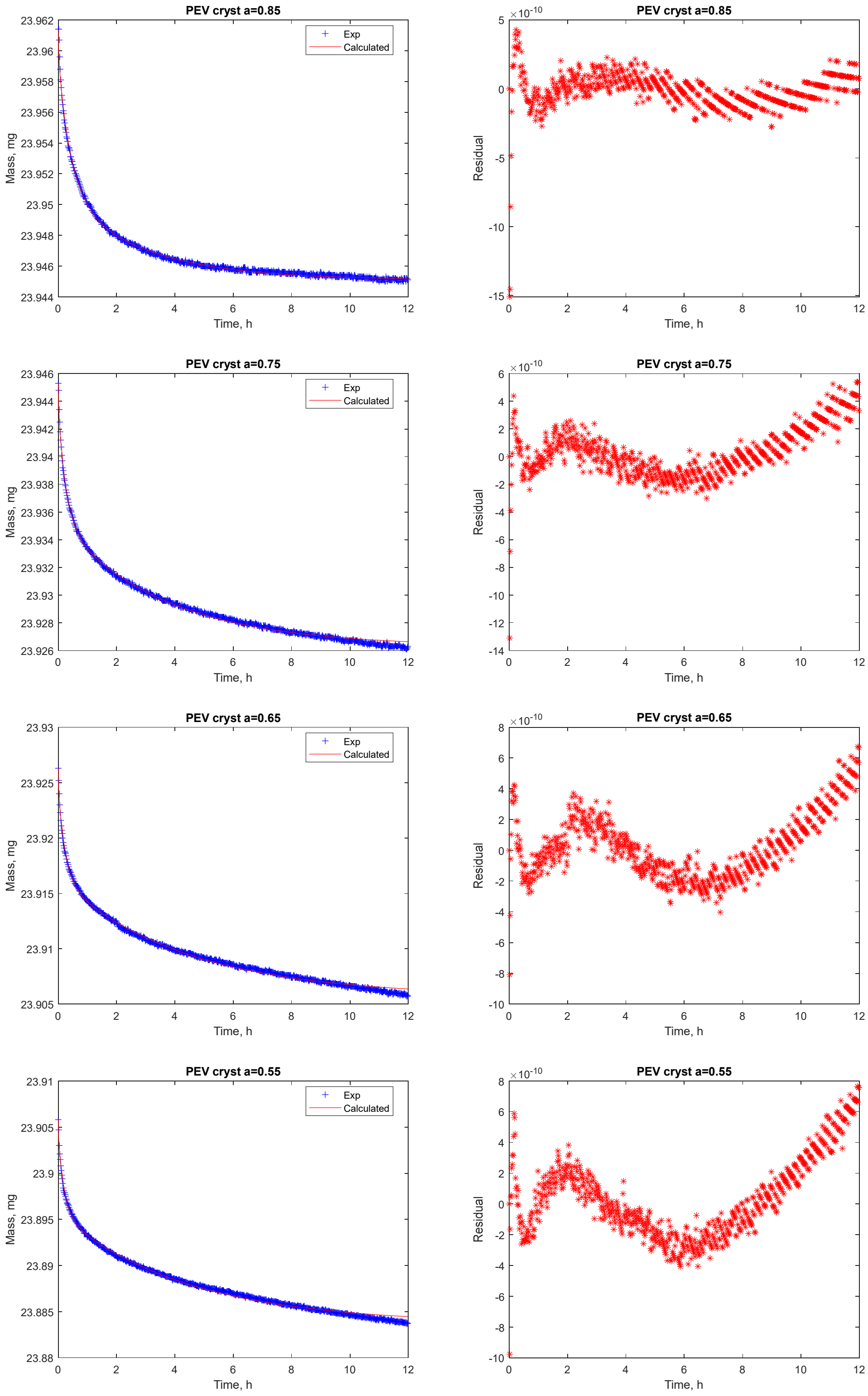
PEV cryst a=0.85
PEV cryst a=0.75
PEV cryst a=0.65
PEV cryst a=0.55
Exp
Calculated
Mass, mg
Residual
Time, h
×10^-10

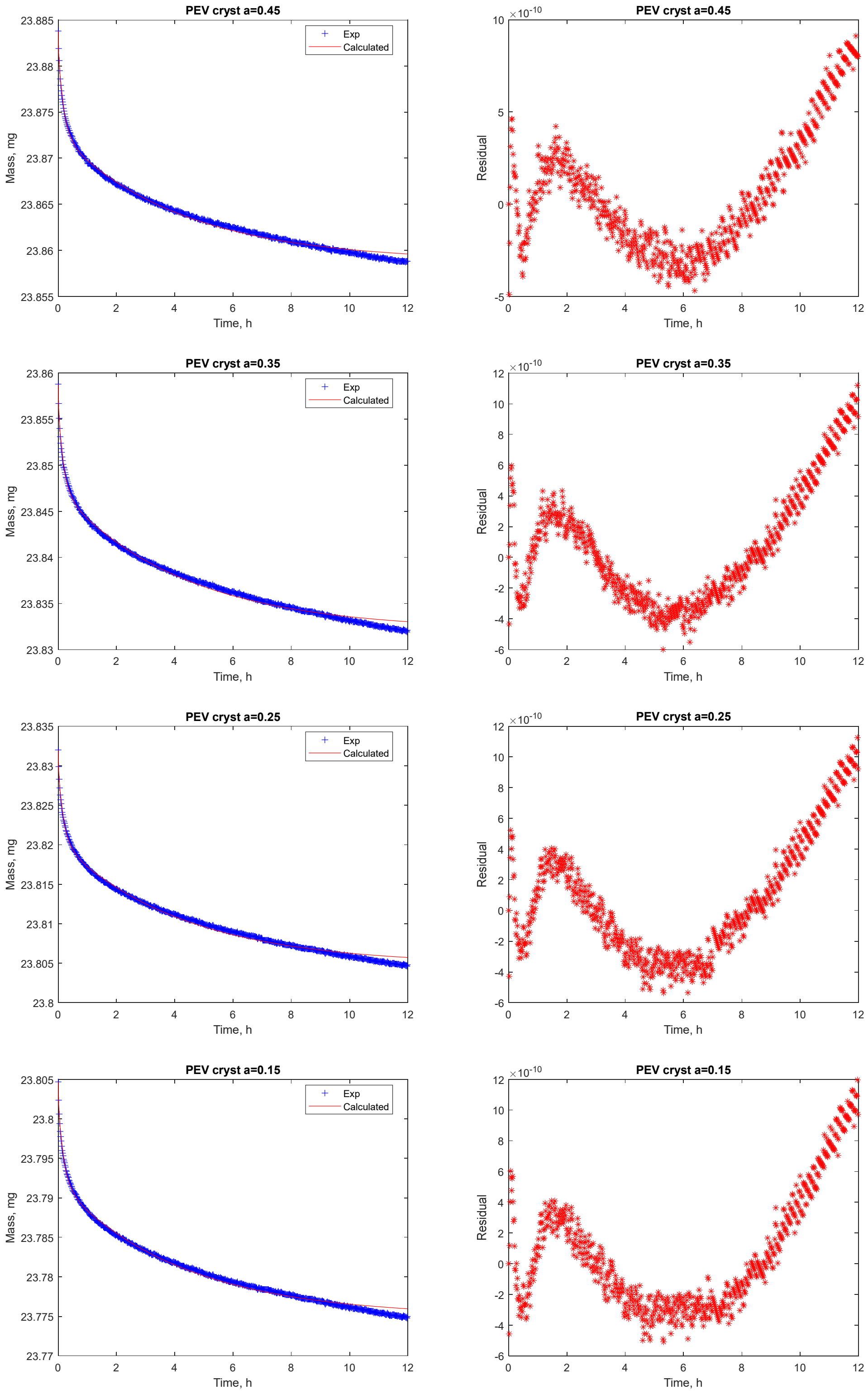
PEV cryst a=0.45
Exp
Calculated
Mass, mg
Time, h
PEV cryst a=0.45
Residual
Time, h
PEV cryst a=0.35
Exp
Calculated
Mass, mg
Time, h
PEV cryst a=0.35
Residual
Time, h
PEV cryst a=0.25
Exp
Calculated
Mass, mg
Time, h
PEV cryst a=0.25
Residual
Time, h
PEV cryst a=0.15
Exp
Calculated
Mass, mg
Time, h
PEV cryst a=0.15
Residual
Time, h

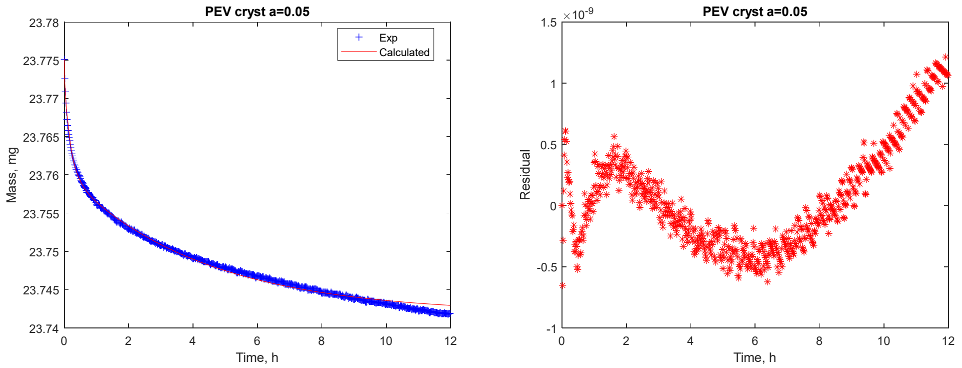

Figure S 4. A) Kinetic sorption/desorption data for water vapor on semicrystalline PEV at 20 °C measured by DVS. Red lines represent model fits from the BH model while experimental data are represented in blue. **B)** Root mean square errors between experimental sorption kinetic data m_{exp} and the simulated ones m_{sim} according to BH model fit.
The sorption intervals are labelled in each respective graph.

The red lines labelled as "calculated" represent the BH model fits from Equation 7 while the experimental data are reported in blue.

For amorphous PEV, the BH model fits well the kinetic curves at low water vapor activity while at high a_w the model does not describe very accurately the experimental data but still acceptable (averaged RMSE = 1.08×10^{-8} mg over the entire a_w interval). For semicrystalline PEV, the BH model fits well all the kinetic curves (RMSE = 0.79×10^{-8} mg). The kinetic curves for whose the fitting is unacceptable have not been considered for diffusivity determination.
